# Multisystem cytomegalovirus end-organ disease in a patient with advanced HIV

**DOI:** 10.4102/sajid.v37i1.468

**Published:** 2022-11-11

**Authors:** Ruan Spies, Pierre Joubert, Dharshnee Chetty, Sipho Dlamini, Muhammed S. Moosa

**Affiliations:** 1 Department of Medicine, New Somerset Hospital, Cape Town, South Africa; 2 Division of Anatomical Pathology, Department of Pathology, Faculty of Health Sciences, University of Cape Town and Groote Schuur Hospital, Cape Town, South Africa; 3 Division of Infectious Diseases and HIV Medicine, Faculty of Health Sciences, University of Cape Town, Cape Town, South Africa; 4 Department of Medicine, Faculty of Health Sciences, University of Cape Town, Cape Town, South Africa

**Keywords:** cytomegalovirus, AIDS, HIV, CMV, end-organ disease, opportunistic infection

## Abstract

Cytomegalovirus (CMV) infection is common in people living with HIV, but multisystem CMV end-organ disease (EOD) is rare following the introduction of effective antiretroviral therapy. We present the case of a patient with advanced HIV and multisystem manifestations of CMV EOD.

**Contributions:**

This case report highlights the potential morbidity and mortality associated with CMV disease in patients with advanced HIV. Clinicians should be vigilant in considering CMV EOD in patients with advanced HIV and visual, neurological and gastointestinal symptoms.

## Introduction

Cytomegalovirus (CMV) infection is common in people living with HIV; however, the incidence of CMV end-organ disease (EOD) has reduced dramatically following the introduction of effective antiretroviral therapy (ART).^1^ The multisystem complications of CMV EOD are well described; however, only a few case reports can be found in the literature which describe patients with simultaneous multisystem CMV EOD.^2,3^ In this case report, we describe a patient with advanced HIV and multiple end-organ manifestations of CMV disease.

## Patient presentation

A 30-year-old man was presented to a regional hospital in Cape Town, South Africa, with a 3-month history of odynophagia, vomiting, abdominal pain and unilateral vision loss. He reported no respiratory complaints or diarrhoea. He had been diagnosed with HIV and drug-sensitive tuberculosis (TB) six months earlier and was initiated on tenofovir, lamivudine and dolutegravir (as well as the first-line anti-TB treatment). He admitted to interrupting ART and anti-TB treatment because of gastrointestinal symptoms. He gave a history of being diagnosed with ataxia of undetermined cause by a neurologist three months earlier. The workup for his ataxia, which included serum thyroid stimulating hormone, vitamin B12 levels, cerebrospinal fluid analysis, and magnetic resonance imaging (MRI) of his brain and spine, was normal.

On examination, he looked chronically ill with muscle wasting and conjunctival pallor. His vital signs were within normal limits, and his abdominal examination revealed epigastric tenderness but no guarding or organomegaly. On neurological assessment, he was alert and orientated with no neck stiffness. He had cerebellar signs that included truncal ataxia, bilateral intention tremor, dysdiadochokinesis and positive heel-shin tests bilaterally. Examination of visual acuity demonstrated no light perception in the left eye and 6/6 vision in the right eye.

**FIGURE 1:**
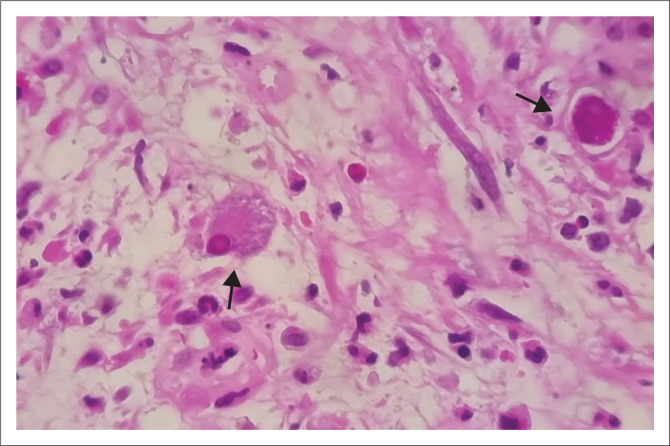
Haematoxylin and eosin (H&E) stain of the oesophageal lesion (at × 40 magnification) demonstrating cytomegalovirus infected cells with intra-nuclear cytomegalovirus inclusions (black arrows).

**FIGURE 2:**
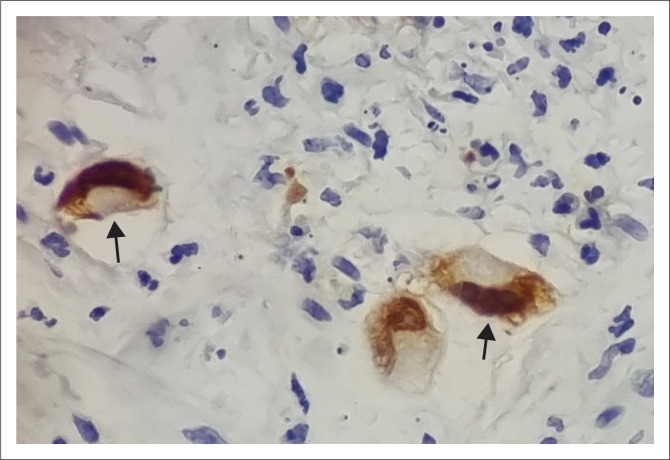
Immunohistochemistry for cytomegalovirus (at × 40 magnification) demonstrating intra-nuclear inclusions (black arrows).

A urine lipoarabinomannan assay (LAM) for TB was negative, and the chest radiograph was unremarkable. Direct ophthalmoscopy demonstrated areas of retinal haemorrhage and necrosis in the left eye. These features were confirmed by an ophthalmologist to be in keeping with CMV retinitis. He had a pancytopenia with neutropenia (Table 1). Vitamin B12, folate and haptoglobin were normal, and the peripheral smear did not demonstrate any features of haemolysis. Renal function and liver enzymes were normal. His CD4 count was 5 cells/μL and his serum CMV viral load was elevated at 1 322 783 IU/mL (Log 6.1). Cerebrospinal fluid (CSF) analysis revealed normal glucose at 2.9 mmol/L, raised protein at 0.76 g/L, no cells and an elevated CMV viral load at 481 617 IU/mL (Log 5.7). CSF microscopy and culture, cryptococcal antigen and GeneXpert Ultra testing were negative. An abdominal ultrasound showed no evidence of opportunistic infections. Gastroscopy demonstrated severe oesophageal candida, pangastritis and a cherry-red papule at the gastroesophageal junction. Biopsy and histology of the lesion showed acute inflammation, focally ulcerated mucosal fragments with intra-nuclear CMV inclusions (Figure 1 and Figure 2). Kaposi’s sarcoma was excluded on histology and by a negative HHV-8 immunohistochemical stain.

**TABLE 1:** Full blood count and absolute neutrophil count at presentation and 1 week into ganciclovir therapy.

Laboratory value	Normal range	At presentation	1 week into ganciclovir
**White cell count (×10^9^/L)**	3.92–10.40	1.33	0.36
**Absolute neutrophil count (×10^9^)**	1.40–4.20	0.73	0.1
**Haemoglobin (g/dL)**	13.0–17.0	6.6	5.3
**Mean corpuscular volume (fL)**	83.1–101.6	90.5	86.6
**Platelets (×10^9^/L)**	171–388	136	238

Our patient was diagnosed with multisystem CMV EOD: retinitis, encephalitis and oesophagitis, with advanced HIV-related immunosuppression. Intravenous (IV) ganciclovir, IV fluconazole and IV pantoprazole were initiated, together with his previous ART. He developed worsening cytopenia on ganciclovir (Table 1) and was transferred to a tertiary referral hospital for further investigation, including neuroimaging, and management. Our patient died three days following his transfer, with the precise cause of death being unknown.

## Discussion

Cytomegalovirus is an enveloped double-stranded DNA virus in the herpes virus family with a high seroprevalence in South Africa.^4^ Cytomegalovirus infection is defined by the detection of viral antigen or nucleic acid in a tissue or body fluid (regardless of symptoms and signs), while CMV EOD describes the syndrome of organ dysfunction resulting from CMV infection. Infection is usually asymptomatic in immunocompetent individuals but may result in viremia and EOD in people with advanced immunosuppression, including people living with HIV with CD4 counts < 50 cells/μL.^1^

Cytomegalovirus retinitis is the most common presentation of CMV EOD, with the gastrointestinal tract, lower respiratory tract and central nervous system representing other typical sites of EOD.^1^ There are no studies that report on the global epidemiology of CMV EOD; however, the prevalence rate of CMV EOD in Africa has been estimated, from small African cohorts of people living with HIV, to be 0.5% – 14.0%.^5,6,7^ A larger cohort study of 400 people living with HIV and CD4 count < 100 cells/μL from India described a prevalence rate of 40.5%; only six patients had evidence of CMV EOD in more than two systems.^8^ To our knowledge, simultaneous multisystem CMV EOD is uncommonly diagnosed in living patients. However, a review of an African autopsy series of people living with HIV and advanced immunosuppression identified CMV disease in 4.0% – 18.0% of cases, with multi-organ disease being common amongst these.^9^ This discrepancy between antemortem and post-mortem reports of CMV EOD raises two questions: (1) Does subclinical multisystem CMV infection occur more commonly than expected, and (2) Is confirming CMV EOD diagnostically challenging – firstly, because of the overlapping clinical features of CMV EOD with other opportunistic infections and secondly, because of difficulties in obtaining tissue specimens for confirmatory CMV testing.

The neurological presentation of CMV as an ataxic cerebellar syndrome in our patient is also uncommon. Cytomegalovirus neurological disease most commonly presents as a diffuse encephalitis with insidious neuropsychological symptoms progressing to motor, sensory and cranial nerve deficits.^10^ Cytomegalovirus neurological disease may also present as a myelitis, with or without radiculopathy.^10^ CSF findings in CMV encephalitis are non-specific but elevated protein and pleocytosis (which may be either lymphocytic or polymorphonuclear) are typical.^10^ Our patient’s CSF was acellular which is not an uncommon finding. A recent cohort study described an absence of CSF pleocytosis in 22% of patients with CMV encephalitis.^11^

Serological testing for CMV is not specific for the diagnosis of CMV EOD as local seroprevalence is high.^4^ Similarly, detection of CMV viraemia by antigen testing, culture or polymerase chain reaction (PCR) has a poor positive predictive value (PPV) for detecting EOD, with a recent study describing a PPV of 14% – 19% depending on the CMV viral load cut-off used.^12,13^ The diagnosis of CMV EOD, therefore, requires a compatible clinical presentation in conjunction with confirmation of the virus histologically, using molecular testing or culture. An exception is CMV retinitis which is associated with pathognomonic retinal changes on ophthalmoscopy, namely, the ‘pizza pie’ appearance representing areas of confluent retinal necrosis and haemorrhage.^14^

Treatment options for CMV disease include the intravenous antiviral ganciclovir and the oral prodrug, valganciclovir. In CMV retinitis, ganciclovir can also be administered intravitreally.^12^ Foscarnet and cidofovir are second-line antivirals used in ganciclovir-resistant CMV disease or when ganciclovir is contra-indicated. They are only available intravenously, and their use is associated with significant renal toxicity.^12^

## Conclusion

Multisystem CMV EOD in people living with HIV is uncommon; however, it may result in severe morbidity and mortality. Clinicians should, therefore, be vigilant in considering multisystem CMV disease in people living with HIV with advanced immunosuppression who present with concurrent visual, neurological and gastrointestinal symptoms. Although effective treatment options are available, these may be associated with significant toxicity.
